# Kawasaki disease in neonates: a case report and literature review

**DOI:** 10.1186/s12969-024-00959-3

**Published:** 2024-01-29

**Authors:** Mingjun Shen, Die Liu, Fang Ye, Jing Zhang, Jun Wang

**Affiliations:** 1 Department of Clinical Medicine, Beijing University of Chinese Medicine, Beisanhuan East Road, Chaoyang District, 100029 Beijing, China; 2 Department of Pediatrics, China-Japan Friendship Hospital, 2 Yinghuayuan East Street, Chaoyang District, 100029 Beijing, China

**Keywords:** Kawasaki disease, Afebrile, Diagnosis, Neonate, Newborn

## Abstract

**Background:**

Kawasaki disease (KD) is an acute systemic vasculitis of unknown etiology that affects infants and young children but is extremely rare in neonates, especially afebrile KD. We present a case of KD without fever in a neonate and review the literature on KD in neonates.

**Case presentation:**

A newborn female was hospitalized because her peripheral blood leukocytes increased for half a day. The admission diagnosis was considered neonatal sepsis and bacterial meningitis. She had no fever since the admission, but a rash appeared on her face by the 7th day. On day 11 after admission, there was a desquamation on the distal extremities. On day 15 after admission, ultrasound showed non-suppurative cervical lymphadenopathy. Echocardiogram revealed coronary artery aneurysms in both sides. Finally, the patient was diagnosed with incomplete KD (IKD). The follow-up echocardiogram showed that the internal diameter of both coronary arteries returned to normal three months after birth.

**Conclusions:**

Fever, rash, and distal extremity desquamation during the recovery phase are the most common symptoms of IKD. When newborns present with clinical manifestations such as rash, distal extremity desquamation and cervical lymph adenitis and with an increased peripheral blood leukocyte count and progressive increase in platelets simultaneously, the medical staff should be highly alert to the possibility of KD even without fever. The echocardiogram needs to be performed promptly. The incidence of coronary artery lesions is significantly higher if neonatal KD patients miss timely diagnosis and treatment.

## Background

Kawasaki disease (KD) is an acute systemic vasculitis of unknown etiology that affects infants and young children [1], but is extremely rare in neonates, especially afebrile KD. Data on 130,323 patients from the Japanese nationwide surveys of KD (2001–2012) identified 23 neonatal KD cases, representing 0.02% of KD in patients of all ages [2]. In this study, we present a neonatal case of incomplete KD (IKD) without fever and review the literature on KD in neonates. This report aims to increase awareness of afebrile KD in neonates to reduce the risk of cardiac complications.

## Case presentation

A 19-hour-old female patient was transferred to the pediatric ward of China-Japan Friendship Hospital because her peripheral blood leukocytes increased for half a day. She was G1P1, at a gestational age of 40 weeks + 2days, and delivered by cesarean section due to acute intrauterine distress with III-degree contaminated amniotic fluid and slowed fetal heart rate. The Apgar score was 10-point at 1, 5, and 10 min after birth, respectively. The birth weight was 3386 g. The child had no fever, no irritability with a high-pitched cry, no convulsions, and no groans or vomiting after admission. Her temperature was 36.5 ℃, pulse 140/minute, respiration 40/minute, BP 80/55 mmHg, capillary refill less than two seconds. The face and trunk were light yellow. The bregma was bulged and the pressure was slightly higher. The neck resistance was suspiciously positive. The muscular tension in the limbs was normal, and the primitive reflexes were derived.

Laboratory investigations on the day of birth showed white blood cell (WBC) was 41.33 × 10^9^/L (neutrophils 75.3%, lymphocytes 15.4%, monocytes 7.8%), red blood cell (RBC) was 4.65 × 10^12^/L, hemoglobin was 167 g/L, platelet (PLT) was 266 × 10^9^/L, and C-reactive protein (CRP) was 6.86 mg/L. Total bilirubin was 124.11 µmol/L and direct bilirubin was 10.4 µmol/L. The cerebrospinal fluid contained total cells was 15 × 10^6^/L, WBC was 13 × 10^6^/L (multinuclear 85%, mononuclear 15%), protein 0.603 g/L, glucose 4.48 mmol/L (peripheral blood glucose: 5.1 mmol/L), and LDH 96 IU/L. Hepatitis B virus, hepatitis C virus, human immunodeficiency virus, treponema pallidum antibody and TORCH were all negative. Rheumatoid factor was normal. Chest X-ray, cranial ultrasound and abdominal ultrasound were unremarkable. Amplitude integration EEG showed no abnormal discharges. The patient was suspiciously diagnosed with neonatal sepsis and bacterial meningitis upon admission. Meropenem and vancomycin were given to control the infection. Mannitol was used to lower the intracranial pressure, and dexamethasone was used to prevent adhesions. On day 2 of hospitalization, intravenous immunoglobulin (IVIG) was used for three days (total dose 2 g/kg) as supportive therapy.

On day 4, cerebrospinal fluid was rechecked and contained total cells 6 × 10^6^/L, WBC 5 × 10^6^/L, protein 0.758 g/L, glucose 2.21 mmol/L (peripheral blood glucose 4.9 mmol/L), and LDH 76 IU/L. She had transient hyponatremia (127 mmol/L). Bacterial cultures from blood and cerebrospinal fluid were sterile.

**Fig. 1:**
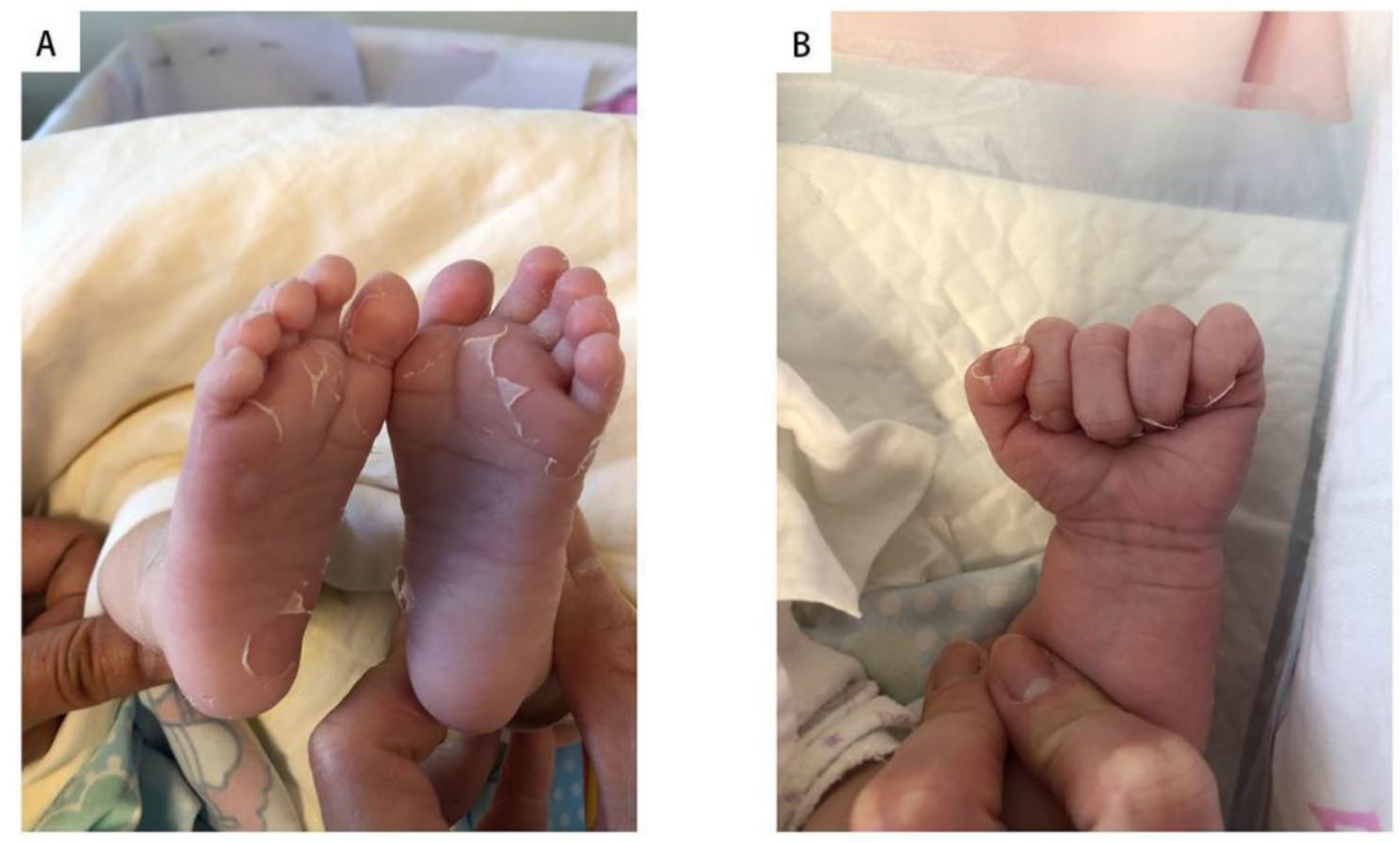
Sheet-like desquamation of extremities emerged on day 11. (**A**) Skin on toes peeling. (**B**) Skin on fingers peeling

**Fig. 2:**
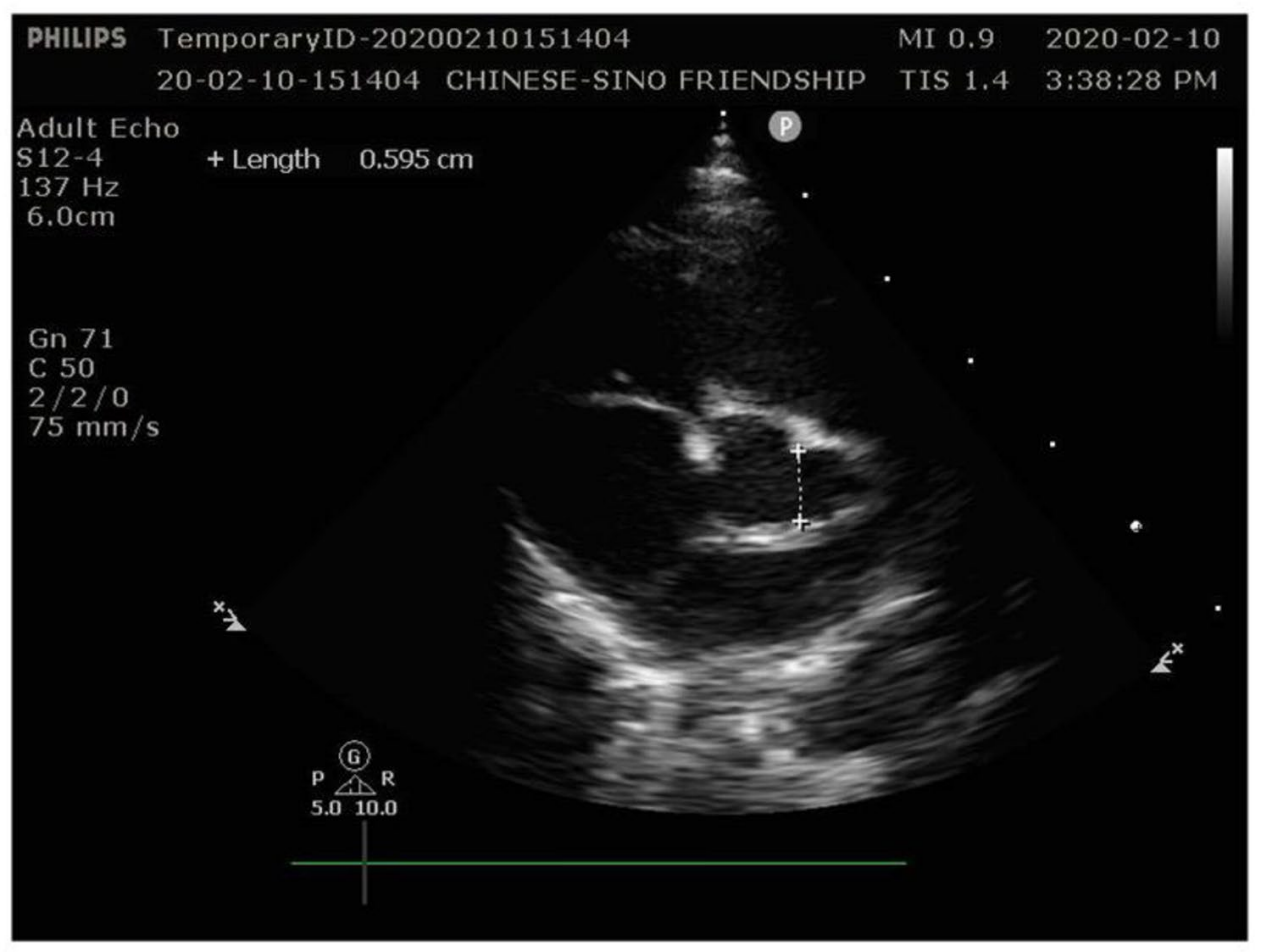
Echocardiography on 15th day after birth revealed coronary artery aneurysms in the left coronary artery

The patient had no fever since admission, but a rash appeared on her face by the 7th day and lasted for five days. The PLT reached from 603 × 10^9^/L on day 7 to 1345 × 10^9^/L on day 12 (Table 1). Distal extremity desquamation began on day 11 and continued for ten days (Fig. 1). However, other manifestations including conjunctivitis, erythematous dry lips, red raspberry tongue and swollen extremities did not appeared. Low molecular dextran was given to reduce blood viscosity, enoxaparin sodium was given for anticoagulation, and dipyridamole and low-dose aspirin (5 mg/kg) were administered for anti-platelet aggregation. On the 15th day of the illness, ultrasound showed non-suppurative cervical lymphadenopathy. Echocardiogram showed that the internal diameter of the proximal segment of the left main coronary artery (LMCA) was 5.9 mm (Z = 11.40), the internal diameter of the left anterior descending coronary artery (LAD) was 2.4 mm (Z = 5.19) and the internal diameter of the right coronary artery (RCA) was 3.1 mm (Z = 8.07) (Fig. 2). Electrocardiogram was normal. Moreover, no abnormal blood flow was found in the arteries of the upper and lower extremities. No thrombus was found in the deep veins. Finally, the patient was diagnosed with IKD. Low-dose aspirin and dipyridamole were given to prevent platelet aggregation continuously.

**Table 1:** Routine blood examination of the patient

Age	WBC ×10^9^/L	N%	L%	RBC ×10^12^/L	Hb g/L	PLT ×10^9^/L	CRP mg/L	PCT ng/ml
Day 0	41.33	75.3	15.4	4.65	167	266	6.86	
Day 1	33.16	74	16.8	3.94	141	291	5.9	
Day 2	25.59	72.5	17.5	3.56	128	316	< 2.5	
Day 4	17.28	63.7	20.5	3.58	129	171	< 2.5	0.2
Day 7	20.25	56.7	27.8	3.03	108	603	< 2.5	< 0.1
Day 12	25.78	59.2	30.4	3.14	112	1345	< 2.5	0.12
Day 14	14.84	27.8	56.2	2.9	102	1010	< 2.5	0.16
Day 16	11.69	27.5	44.6	3.01	105	971	< 2.5	0.49
Day 19	10.22	13.7	57.7	2.75	94	710	< 2.5	
Day 21	10.17	17.2	61.2	2.69	90	499	< 2.5	
5 weeks	13.42	30.1	52.6	3.07	101	579	< 2.5	
7 weeks	10.76	47.4	31.0	4.40	116	319	< 2.5	
12 weeks	9.95	27.2	56.2	3.15	100	524	4.12	

WBC: White blood cell; N: Neutrophils; L: Lymphocytes; RBC: Red blood cell; Hb: Hemoglobin; PLT: Platelet; CRP: C-reactive protein; PCT: Procalcitonin

On 21 days after admission, the blood analyses showed WBC was 10.17 × 10^9^/L (neutrophils was 17.2%, lymphocytes was 61.2%, monocytes was 12.1%), RBC was 2.69 × 10^12^/L, hemoglobin was 90 g/L, PLT was 499 × 10^9^/L, and CRP was < 2.5 mg/L. Echocardiogram revealed LMCA of 2.6 mm (Z = 4.29), LAD of 2.1 mm (Z = 4.13), and RCA of 2.3 mm (Z = 4.95).

The patient was discharged with low-dose aspirin and dipyridamole after 21 days in the hospital. Regular follow-up was carried out every 2–4 weeks after discharge. Echocardiogram revealed LMCA of 2.0 mm (Z = 1.69) and RCA of 1.7 mm (Z = 1.78) three months after birth (Table 2).

**Table 2:** Coronary artery parameters of the patient

Age	LCA (mm)	LAD(mm)	RCA (mm)
Day 15	5.9 (Z = 11.4)	2.4 (Z = 5.19)	3.1 (Z = 8.07)
Day 21	2.6 (Z = 4.29)	2.1 (Z = 4.13)	2.3 (Z = 4.95)
12 weeks	2.0 (Z = 1.69)		1.7 (Z = 1.78)

LCA: Left coronary artery; LAD: Left anterior descending; RCA: Right coronary artery

## Discussion

KD is an acute febrile condition seen in children. The diagnostic criteria for KD are fever, bilateral bulbar conjunctival injection, changes in the lips and oral cavity, rash, changes in the peripheral extremities, and non-suppurative cervical lymphadenopathy. Fever is no longer necessary for the diagnosis of KD, according to the sixth revised edition of the Japanese diagnostic criteria [3]. Statistically, neonates with KD have a higher risk of incomplete presentation than older children [2].

The number of IKD cases increased yearly from 10% to the current level, which is greater than 20% of all KD patients [3]. Fever, redness, and swelling of the extremities are the most common symptoms of IKD [4]. We searched case reports of neonatal KD published in English from January 1, 2000 to February 18, 2023 with the search formula: ((“Mucocutaneous Lymph Node Syndrome“[Mesh]) OR (((Kawasaki Syndrome[Title/Abstract]) OR (Lymph Node Syndrome, Mucocutaneous [Title/Abstract])) OR (Kawasaki Disease [Title/Abstract]))) AND ((“Infant, Newborn“[Mesh]) OR (((((((((Infants, Newborn) OR (Newborn Infant)) OR (Newborns)) OR (Newborn)) OR (Neonate)) OR (Neonates)). The inclusion criteria were cases of KD in newborns and the diagnosis met the *Revision of diagnostic guidelines for Kawasaki disease (6th revised edition)* [3]. The exclusion criteria were duplication or literature with incomplete case information (no clinical features, no laboratory findings and outcomes). Nineteen cases in 15 papers were analyzed [2, 5–18] (Table 3). IKD accounted for 68.4% (13/19). The clinical manifestations included rash in 94.7% (18/19), changes in the terminal extremities in 78.9% (15/19), fever in 78.9% (15/19), erythematous changes in the lips and oral mucosa in 68.4% (13/19), bilateral non-purulent conjunctivitis in 42.1% (8/19), cervical lymph adenitis in 10.5% (2/19), and coronary artery lesions (CALs) in 89.5% (17/19). Laboratory tests showed that elevated CRP accounted for 63.2% (12/19) and PLT > 300 × 10^9^/L accounted for 57.9% (11/19) (Table 4).

**Table 3:** Summary of the case reports of neonatal Kawasaki disease

Case	Ref	Diagnosis	Gender	Age at onset(days)	Fever duration(days)	Rash	Conjunctival congestion	Oral changes	Extremity edema	Cervical lymph adenitis	Peeling	Coronary artery lesions	PLT	CRP	ESR	IVIG response	CAL outcome(last F/U)
1	PR	IKD	F	0	-	+	-	-	-	+	+	+	H	H	UN	+	N(12 weeks)
2	5	KD	M	24	12	+	-	+	+	+	+	+	H	H	H	+	N(1 year)
3	6	KD	F	13	7	+	+	+	+	-	+	+	L	H	UN	-	AB(80 days)
4	7	IKD	M	14	18	+	+	-	-	-	+	+	H	H	H	+	AB(7 months)
5	8	IKD	M	15	5	+	+	-	+	-	-	+	H	N	N	+	N(1 year)
6	9	IKD	M	5	-	+	+	+	+	-	+	+	H	H	H	+	N(14 months)
7	10	IKD	M	19	-	+	+	+	+	-	+	-	UN	H	UN	+	N
8	2	IKD	F	22	4	+	-	-	+	-	+	+	N	H	N	+	N(3 months)
9	11	IKD	M	1	-	-	-	-	-	-	-	+	L	UN	N	Not use	N(1 year)
10	12	IKD	F	21	4	+	-	+	+	-	UN	+	UN	N	N	+	N(6 weeks)
11	12	IKD	F	14	3	+	-	+	+	-	+	+	N	N	N	+	N(6 months)
12	12	IKD	M	16	4	+	-	+	+	-	UN	-	N	N	N	+	N
13	13	IKD	M	18	6	+	-	+	+	-	+	+	H	N	UN	+	N(6 weeks)
14	13	IKD	M	16	9	+	-	+	+	-	UN	+	H	H	UN	+	N(11 years)
15	14	IKD	M	8	> 9	+	-	-	-	-	+	+	H	H	H	+	AB(9 weeks)
16	15	KD	F	8	9	+	+	+	+	-	+	+	N	H	UN	+	N(6 weeks)
17	16	KD	F	16	13	+	+	+	+	-	UN	+	H	H	H	+	UN
18	17	KD	F	20	UN	+	-	+	+	-	+	+	H	H	N	+	AB(8 weeks)
19	18	KD	M	10	5	+	+	+	+	-	+	+	H	N	UN	+	AB(2.5years)

AB: Abnormal; CAL: Coronary artery lesions; CRP: C-reactive protein; ESR: Erythrocyte sedimentation rate; F: Female; H: High; IKD: Incomplete Kawasaki disease; KD: Kawasaki disease; L: Low; N: Normal; PCT: Procalcitonin; PR: Present report; UN: Unknown

**Table 4:** Frequency of clinical and laboratory features of 19 neonatal cases of KD

Clinical and Laboratory Features	Frequency	(%)
IKD	13/19	68.4%
Male	11/19	57.9%
Fever (any duration)	15/19	78.9%
Duration of fever ≥ 5 days	10/19	53%
Polymorphous rash	18/19	94.7%
Extremity changes	15/19	79%
Oral changes	13/19	68.4%
Peeling	13/19	68.4%
Conjunctival congestion	8/19	42.1%
Cervical lymph adenitis	2/19	10.5%
Coronary artery lesion	17/19	89.5%
CRP elevation	12/19	63.2%
Increased PLT count	11/19	57.9%
Increased ESR	5/19	26.3%
IVIG response	17/19	89.5%
CA outcome(last N)	12/19	63.2%

There were four patients with no fever among the 19 neonatal KD cases in our literature review. Only one afebrile patient was diagnosed as IKD purely based on CALs [11]. The other three afebrile patients [9–10], including the present case, had the same clinical manifestations, such as rash and periungual desquamation. In addition to the above-mentioned manifestations, conjunctival congestion, changes in the lips, and extremity edema were also observed in cases 6 and 7 [9–10] (Table 3).

The immune system of newborns is in a special developmental stage, which might lead to heterogeneity in neonatal KD and explain the higher incidence of IKD in neonates than older children. In this case, the patient did not have fever and other clinical manifestations, such as bilateral bulbar conjunctival injection, changes in the lips and oral cavity, probably associated with the early stage of neonate and the impact of early use of IVIG and dexamethasone.

To date, the etiology of KD is not clear. Previous studies suggested that KD is triggered by an infectious agent based on its occurrence in epidemiological clusters, seasonal variation, and a very low risk of recurrence [19]. Other research suggested that neonatal KD could be associated with sepsis and pneumonia [20, 21]. Although the patient’s blood culture and cerebrospinal fluid were all sterile, the infection could not be excluded since abnormally elevated WBC and III-degree contaminated amniotic fluid at birth. We could not distinguish exactly whether this case was a KD secondary to systemic infection or just a KD case from the beginning.

CALs are the primary serious complication affecting the prognosis of KD. Several studies suggested that infants under the age of 6 months not only present more commonly with IKD, but are also at higher risk for coronary artery abnormalities and death [8]. In the 19 cases of neonatal KD mentioned above, CALs occurred in 89.5% in 19 neonates and 75% in the four cases of afebrile neonatal KD. 61.1% (11/18) of the patients with CALs had a favorable prognosis after using IVIG. Although this patient initially presented with medium to large coronary aneurysms, the internal diameter of the coronary arteries returned to normal after three months by IVIG treatment on 2nd day after birth. The 24th Nationwide Surveillance in Japan reported that approximately 9%, 25%, and 35% of KD patients received the first IVIG treatment on the 3rd, 4th, and 5th days of illness, respectively, and the prevalence of CALs were lower than before [3]. Consistent with this finding, our case suggests that early use of IVIG might be beneficial for long-term prognosis in KD.

## Conclusions

This case report and review of the literature suggest a relatively higher incidence of IKD in neonates. Therefore, when newborns present with rash, terminal changes in the extremities or cervical lymph adenitis, increased peripheral blood leukocyte count and CRP, or progressive increase in platelets, the medical staff should be highly alert to the possibility of KD even without fever. Echocardiogram needs to be performed promptly. The incidence of CALs in neonatal KD is significantly higher. Timely diagnosis and treatment are essential for neonatal KD to improve the prognosis.

## Data Availability

The datasets used and/or analysed during the current study are available from the corresponding author on reasonable request.

## Abbreviations

AB: Abnormal
CAAs: Coronary artery aneurysms
CALs: Coronary artery lesions
CRP: C-reactive protein
ESR: Erythrocyte sedimentation rate
IKD: Incomplete Kawasaki disease
IVIG: Intravenous immunoglobulin
KD: Kawasaki disease
LAD: Left anterior descending
LCA: Left coronary artery
PCT: Procalcitonin
PLT: Platelet
RBC: Red blood cell
RCA: Right coronary artery
WBC: White blood cell
